# Utility Tunnel Settlement Monitoring Using Distributed Fiber Optic and Ground Penetrating Radar Technologies

**DOI:** 10.3390/s25226964

**Published:** 2025-11-14

**Authors:** Jinyong Li, Dingfeng Cao, Tao Xiao, Chunyan Wang

**Affiliations:** 1 Nanping Wusha Expressway Co., Ltd., Jianyang 354200, China; 2 State Key Laboratory for Tunnel Engineering, School of Civil Engineering, Sun Yat-sen University, Zhuhai 519082, China; 3 China MCC20 Group Corp., Ltd., Shanghai 201900, China

**Keywords:** multi-purpose utility tunnel, Brillouin frequency domain analysis (BOFDA), fiber Bragg grating (FBG), ground penetrating radar (GPR), ground settlement

## Abstract

Settlement and deformation of multi-purpose utility tunnels (MUTs) are critical factors affecting their structural integrity and service life; however, effective identification methods remain limited. This study proposes a comprehensive approach integrating Brillouin frequency domain analysis (BOFDA), fiber Bragg grating (FBG), and ground penetrating radar (GPR) technologies, which was successfully applied to an MUT comprising three tanks in Baiyin City, Gansu Province, China. BOFDA enables precise localization of settlement points, FBG-based dislocation meters facilitate posture recognition of the MUT, and GPR is employed for detailed analysis of settlement causes. The results indicate that MUT deformation primarily manifests as displacement at joint locations, supplemented by deformation of the tunnel structure itself. Rotation, even settlement, and uneven settlement were identified through three FBG-based dislocation meters installed on the top and side walls. The primary causes of MUT settlement include mudstone compression and collapse of loess.

## 1. Introduction

The multi-purpose utility tunnel (MUT) was first built in Paris, France in 1850, which integrates all pipelines including electricity, telecommunication, gas, heat, and water or sewage pipes [1]. The MUT has the obvious advantages of being space-saving, reducing maintenance and repair, enhancing safety, and decreasing environmental impact [2,3], which makes it widely accepted. In China, the total length of the MUT has been exceeded to 8500 km by 2021 [4]. However, the MUT also has unavoidable disadvantages such as high initial construction costs, long cost recovery period, and large investment risks. Therefore, it is difficult to promote its use in underdeveloped areas. Compared with pipelines, once problems occur with the MUT, maintenance is more difficult, more costly, and takes a longer construction period. This is because if the MUT structure itself is damaged, it will also affect the various internal pipelines, causing secondary disasters such as fire, gas explosion, and water pipe rupture [2,5]. Therefore, the safety and stability of the MUT is the prerequisite for ensuring the safe operation of the entire internal pipe system [4].

The damage of the MUT structure is mainly caused by the settlement foundation, which can be manifested in the form of regional overall settlement, local uneven settlement, and dislocation or flipping. If the deformation is severe, it may cause stress concentration, convergence, or even cracking [6,7,8]. The deformation of the utility tunnel is also significantly affected by other factors such as fire, explosion, excavations, and earthquakes [9]. The methods for investigating the mechanism of MUT settlement, deformation, and cracking can be divided into three categories, including numerical simulation, model testing, and in situ monitoring.

The numerical method is low-cost and can be used to simulate various harsh external conditions (such as fire and explosion). However, the boundary conditions must be simplified during the calculation process, and it is difficult to reproduce non-continuous media [10,11]. Few public reports on the use of model tests to study the deformation characteristics of the MUT can be found due to the high cost and time consumption [11]. Xu et al. [12] physically simulated the influence of active ground fissures in Xi’an, China, on the stress MUT, discovered the nonlinear deformation feature during the development of ground fissures, and divided the entire deformation process into four stages. Lin et al. [13] proposed two shaped prefabricated MUTs (L-shaped and F-shaped), investigated the impermeability, and found that the maximum water pressure can reach up to 0.45 MPa without leakage. There are many cases of using in situ monitoring technology to study MUT deformation. The traditional deformation monitoring manual ground leveling measurements and the data are collected at multi-year intervals, which are unable to capture short-term deformation information and detect disaster generation processes [14]. To overcome these shortcomings, distributed fiber optic sensing (DFOS) technologies were suggested to monitor tunnel deformation. Zhang and Broere [15] designed a special sensor that can simultaneously capture horizontal joint opening and vertical uneven settlement with an accuracy of sub-millimeters. Zhang et al. [15] conducted a full-scale test to capture the mechanical performance and bearing capacity of the MUT, and collected strain distribution using high-precision DFOS. DFOS has also been successfully used to monitor twin tunnel interaction [16], seasonal joint deformation [14,17,18], tunnel excavation [19], and nearby construction activity [20].

Although the feasibility of using DFOS to monitor MUT has been previously proven through many cases, the application potential needs to be further expanded because the climate, geology, hydrology, economy, and maintenance conditions of MUTs are completely different in different areas [9,21]. DFOS can be further divided into fully and quasi-distributed technologies. The quasi-fully DFOS is used to be developed into various displacement sensors for monitoring joint dislocation and cracking, but its cost is often too high to be used in large scale [14,21]. The cheap fully DFOS is able to capture continuous deformation distribution and profiles, but its maximum strain measurement range is less than 2%, which is too small in many cases. In addition, it is difficult to determine the posture (uneven settlement, flip, and convergence) of the MUT after deformation. Therefore, the best monitoring program is to locate the deformation position using fully DFOS, and then install quasi-fully DFOS for further accurate posture monitoring. However, the effective combination of fully and quasi-fully DFOS has not been reported in MUT monitoring.

The DFOS can accurately record the MUT deformation and provide some deductions which occurred in the lower foundations, such as settlement and emergence of emptiness; therefore, it is difficult to directly provide a decision-making basis for disaster handing only using the DFOS. Therefore, these inferences should be verified by new evidence provided by other nondestructive testing technologies. Ground penetrating radar (GPR) has become one of the most common shallow ground surface geophysical survey methods for infrastructure, which emits electromagnetic wave signals into the structure and detects the echoes from changes in the materials [22,23,24]. GPR is usually used to detect geological structures under buildings, locate man-made structures influencing safety, and wet ground area [22] by transmitting radio wave signals into a structure and detecting echoes produced by the material properties’ changes. However, GPR operation is time-consuming, cannot record real-time data, and the images are always multi-analytical. Therefore, it is difficult for GPR to detect disasters at the early stage of MUT deformation without DFOS data.

Therefore, combining DFOS with GPR is of great significance, as it can simultaneously determine the deformation, posture, and causes of the MUT. After the MUT is constructed, a fully DFOS system is first laid to accurately locate and track the deformation position of the MUT. The sensing optical cable used in the fully DFOS technology is inexpensive, currently priced at approximately USD 2 per meter, and can be widely adopted. The regular monitoring frequency of fully DFOS can be once a quarter or once a month, and the demodulator can be rented, saving managers a significant amount of equipment purchase cost. For most MUTs for most of their length, fully DFOS monitoring results are normal during the operational period, and there is no need to add more monitoring equipment later. When an evolving abnormal strain is detected at a location that happens to be at the joint of the MUT, a quasi-fully DFOS sensor (fiber Bragg grating, FBG) should be deployed at that joint to further monitor the posture changes in the MUT. As the abnormal strain continues to evolve, it is necessary to use GPR to scan the soil at the bottom or outside the side wall of the MUT to confirm the cause of the deformation.

To fill the gap, the DFOS and GPR were used together to determine the deformation and settlement mechanisms of the MUT through a typical case in Baiyin city, Gansu Provience, China. The fully DFOS was used to locate the deformation position, and GPR was used to determine the deformation reason. In addition, a numerical model was established using the finite element method (FEM) to simulate the influence of the size of the cavity in the foundation on the MUT.

## 2. Materials and Methods

### 2.1. Field Site Description

**Figure 1:**
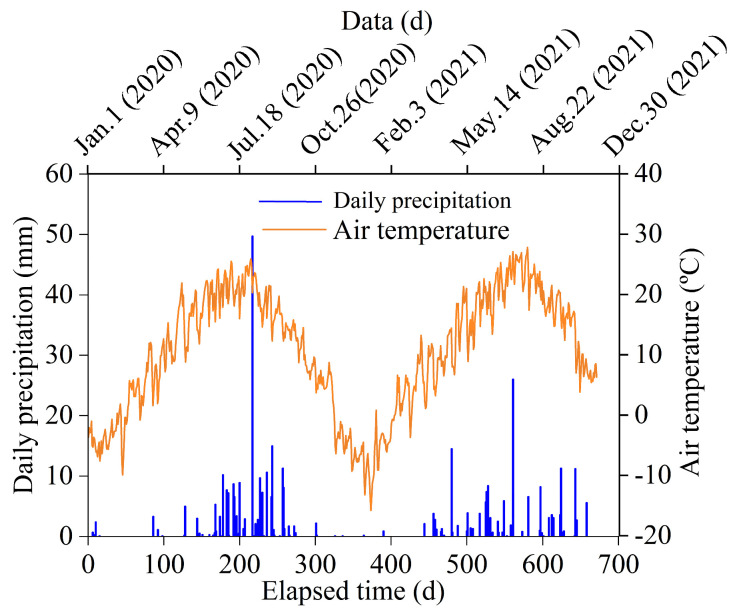
Daily precipitation and air temperature variation in Baiyin, Gansu Province, China.

Baiyin is located in the transition zone among the Tengger Desert, Qilian Mountains, and the Loess Plateau. The climate is semi-arid and arid, and the altitude is between 1275 and 3321 m. There is plenty of sunshine, the annual average temperature is 9.2 °C, and the annual average rainfall is 255.2 mm. However, heavy rainfall during a short period is prone to triggering numerous geological disasters. Figure 1 shows the daily precipitation and air temperature recorded from 1 January 2020 to 20 October 2021.

**Figure 2:**
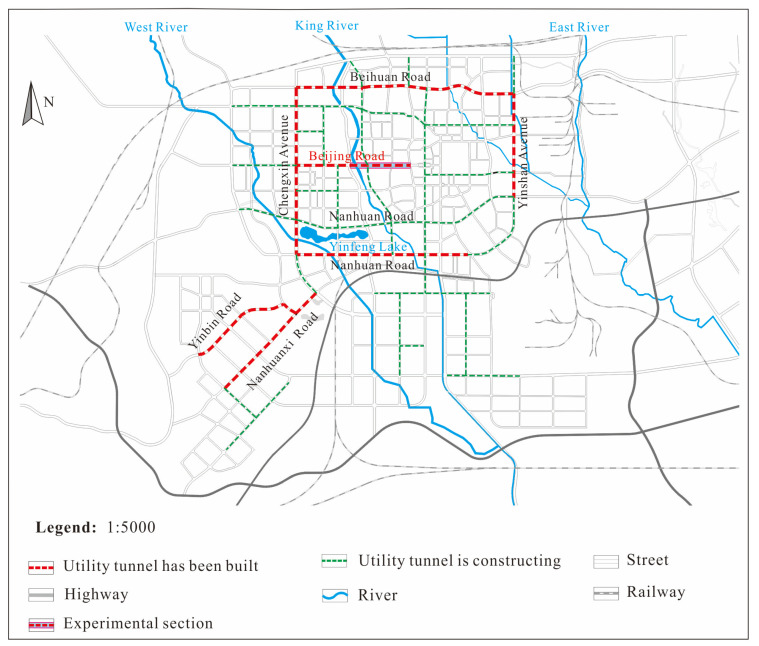
Multiple utility tunnels located in Baiyin.

In April 2015, Baiyin successfully applied to be one of the first national MUT construction pilot cities in China. The public–private partnership model was adopted in this project. The MUTs are constructed under North Ring Road, Yinshan Road, South Ring Road, Chengxin Avenue, Beijing Road, Yingbin Avenue, and South Ring West Road, with a total length of 26.25 km, as shown in Figure 2. Beijing Road is the busiest road with the largest traffic volume; therefore, the MUT beneath this road was selected as the research subject in this study. There are two seasonal rivers running from east to west across Beijing Road. The larger one is named King River, and the smaller one is West River. The landform unit belongs to the tectonic–erosional low mountain. Due to the needs of urban construction, the original landform has been transformed and is now flat and open. The stratigraphic lithology is mainly composed of Quaternary Holocene mixed filled loess, Quaternary Holocene alluvial and diluvial materials, and Cretaceous sandstone.

**Figure 3:**
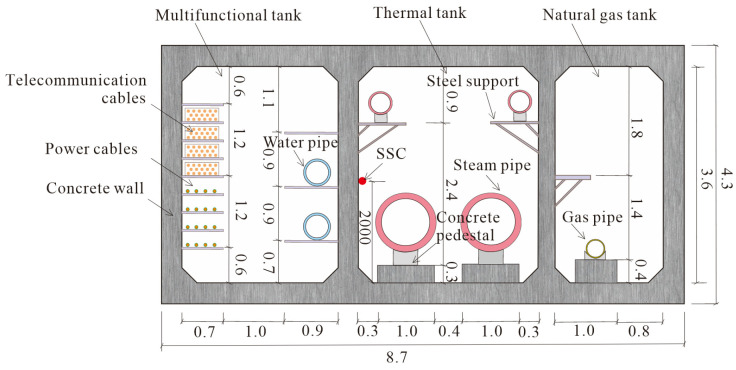
Schematic diagram of cross section and connections of utility tunnel in Baiyin (unit: m).

The cross section and joint connection are shown in Figure 3. Three-compartment rectangular cross section is adopted, which are the natural gas, pipeline, and power tank, respectively, as shown in Figure 3. Four types of pipelines were deployed in MUT, including heat pipe, water supply pipe, gas cables, and power cables.

### 2.2. On-Site Monitoring Scheme Using DFOS and GPR

**Figure 4:**
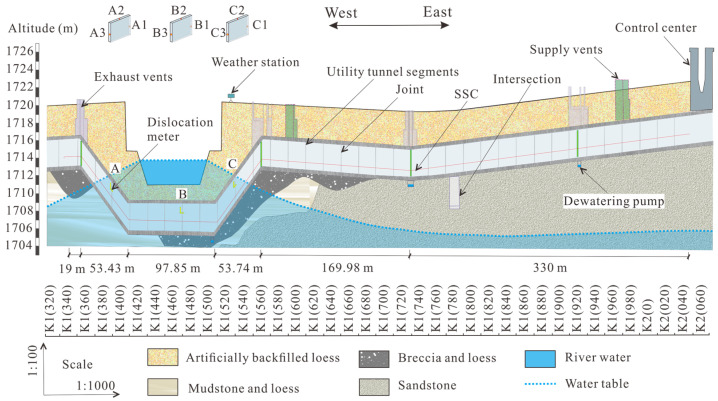
Geological conditions, lithology, and utility tunnel configuration in Beijin Xi Road, Baiyin. A and C are located in the middle of the slope under the river at MUT, while B is located below the center of the river. Subscript 1 is located on the inner wall of MUT in the downstream direction of King River. Subscript 2 is located on the inner side wall of the top of the MUT. Subscript 3 is located on the inner wall of MUT in the upstream direction of King River.

The total length of the investigated MUT is 724 m, and the front section is shown in Figure 4. According to the height change, it can be divided into six sections from left to right, and the length is 19, 53.43, 97.85, 53.74, 169.98, and 330 m. The left four sections cross the King River. There are four exhaust vents, two supply vents, and one control center. A polyurethane sheathed strain sensing cable (SSC) was fixed on the inner wall of the thermal tank with a diameter of 2 mm. Three sections (numbered A, B, and C) beneath the King River were selected to install quasi-fully DFOS dislocation meter. For each section, three positions (two points on the side walls and one point on the top) were selected to install dislocation meters.

**Figure 5:**
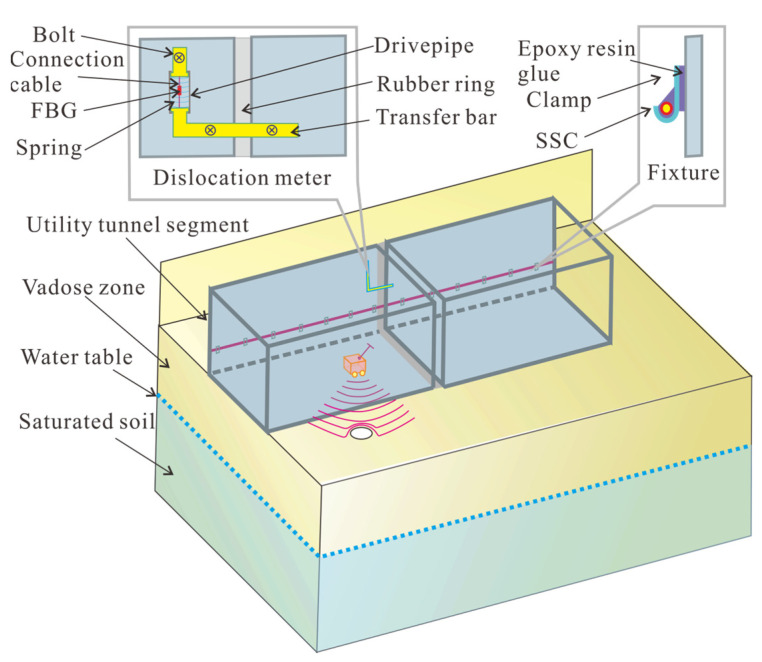
Schematic diagram of utility tunnel detection risk system. GPR is ground penetrating radar. FBG means fiber Bragg grating.

**Figure 6:**
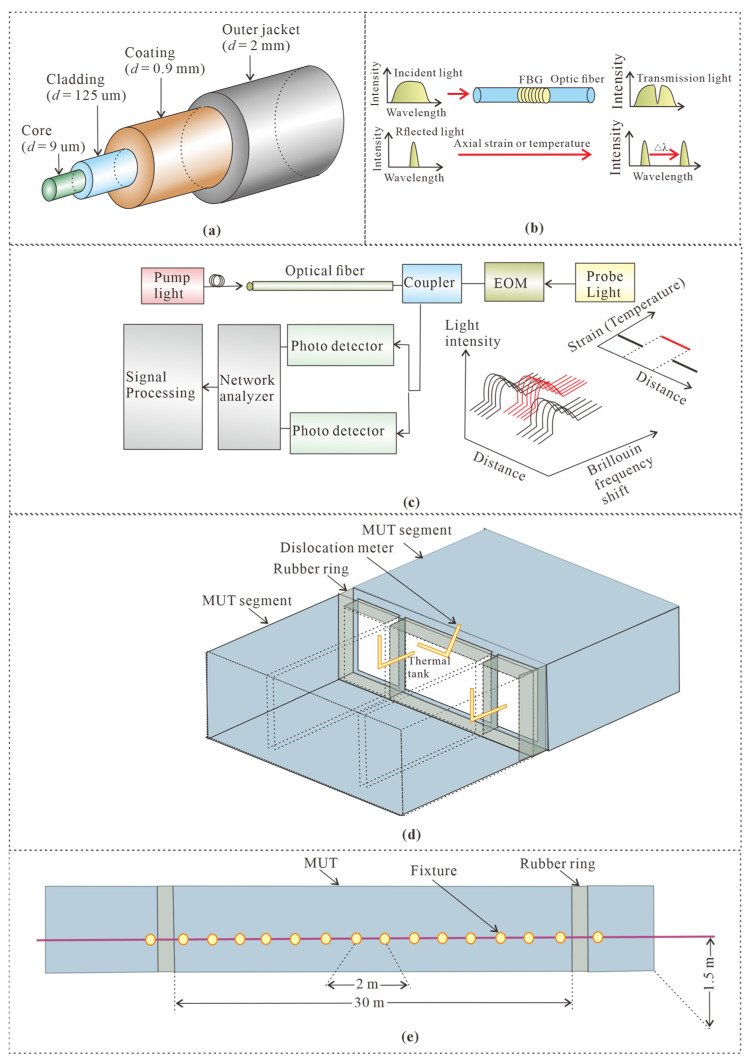
Schematic diagram of fiber optic sensing and sensors installation, (**a**) structure of strain sensing cable, (**b**) working principle of FBG, (**c**) working principle of BOFDA, (**d**) cross section where dislocations are installed, and (**e**) layout of strain sensing cable.

The SSC is fixed on the MUT by clamps, and the distance between adjacent fixture clamps was 2 m. The smooth back of the clamp was glued to the side wall of MUT with a height of 1.5 m, as shown in Figure 5 and Figure 6. There is a slot on the other side of the fixture. After the SSC was inserted into the slot, epoxy resin was used to fill and fix them. The adjacent MUT segment dislocation was monitored by a dislocation meter. The SSC structure, from the inside out, consists of the core, cladding, coating, and outer jacket, as shown in Figure 6a. The length of each MUT segment is 30 m. The dislocation meter was fixed by three active hinge supports that can freely rotate but cannot be moved, as shown in Figure 6d. Therefore, the dislocation displacement between adjacent segments can be converted into the tension or compression deformation of the sparing, and then can be accurately monitored by the FBG sensors. The cavity beneath the MUT was detected by a GPR (SIR-4000, produced by the Geophysical Survey Systems, Nashua, NH, USA). The largest detection depth was 3 m. The starting plane for calculating the maximum exploration depth is the contact surface with the ground penetrating radar. In this study, the ground penetrating radar was dragged along the bottom surface inside the MUT for detection; therefore, the inner surface of the bottom surface of the MUT was taken as the zero-depth position. The SIR 4000 has a matrix 1024 × 768 resolution and 32-bit color.

Five SSC strain data were collected by a Brillouin frequency domain analysis (BOFDA) system (produce by the Suzhou Nanzee Sensing Technology Co., Ltd., Suzhou, China) on 1 January 2020, 12 May 2020, 25 August 2020, 25 November 2020, and 6 January 2021. The data collected on 1 January 2020 were taken as the initial values for the later data subtraction. In addition to these sampling data of SSCs, the dislocation meter was read on 7 December 2021. The GPR data were collected on 6 January 2021.

For the FBG sensors, the ε can be written as [25]

(1) $$\varepsilon =\frac{\Delta \lambda_{B}}{\lambda_{B}\left(1-P_{e}\right)}+\varepsilon_{0}$$

where $\Delta \lambda_{B}$ is the central wavelength of the reflected signal shift (nm), $\lambda_{B}$ is the central wavelength of the reflected signal, and $P_{e}$ is the strain optical coefficient, as shown in Figure 6b. The relationship between stain and Brillouin optical frequency without consideration of temperature variation interference can be expressed as [25]

(2) $$\frac{\varepsilon }{d\varepsilon }=\frac{\upsilon_{B}\left(\varepsilon \right)-\upsilon_{B}\left(0\right)}{d\upsilon_{B}\left(\varepsilon \right)},$$

where ε is strain, $\upsilon_{B}\left(0\right)$ is the Brillouin frequency shift in the initial state (MHz), and $\upsilon_{B}\left(\varepsilon \right)$ is the Brillouin frequency shift at any time (MHz), as shown in Figure 6c. The delamination is inferred by [26]

(3) $$1-R_{l}\left(\theta \right)R_{u}\left(\theta \right)e^{-2\gamma_{1}h\cos\left(\theta \right)}=0,$$

$R_{l}\left(\theta \right)$ and $R_{u}\left(\theta \right)$ are the reflection coefficients at the lower and upper boundaries of the waveguide with an antenna. *h* is the thickness of the waveguide, θ is the angle of incidence, and $\gamma_{1}$ is the propagation constant defined by [26]

(4) $$\gamma_{1}=\frac{j\omega \sqrt{\mu_{b}}}{c_{0}},$$

where $\mu_{b}$ is the real part of the permittivity of layer *b*, and $c_{0}=2.9998\times 10^{8}\;m/s$ is the speed of light in the vacuum.

## 3. Results and Discussion

### 3.1. Settlement and Deformation Detection Base on the Fiber Optic Monitoring

**Figure 7:**
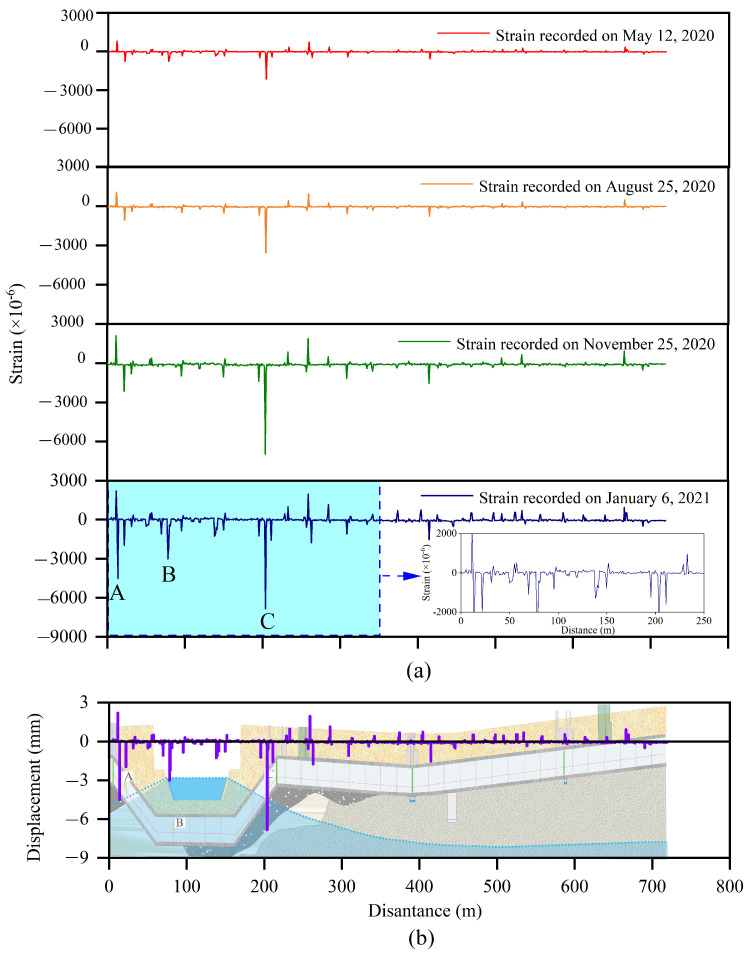
Strain distribution and development along the MUT obtained by the OFDR technology, (**a**) strain, and (**b**) displacement calculated by the strain.

Figure 7a shows the strain distribution recorded by the SSC. The strain curve presents a periodic convex shape, indicating that the deformation is mainly caused by the displacement between the MUT segments, and the deformation of MUT itself can be ignored. Taking the test data collected on 6 January 2021 as an example, the strain at 26 locations was greater than 400 με, which is 68% of the total number of MUTs. Three locations with large strain (>3000 με) correspond to the sections A, B, and C in Figure 4, and the corresponding strains are shown in Table 1. The position numbers with strain larger than 400 με were 5 on 12 May 2020, 6 on 25 August 2020, and quickly reached 18 on 25 November 2020. This strain increase was mainly caused by heavy rainfall on 3 August 2020 (49 mm/d). This heavy rainfall caused the maximum water level of King River to exceed 3 m. From 25 November 2020 to 6 January 2021, the positions with strain larger than 400 με increased from 18 to 26, indicating that the effect of rainfall on the MUT deformation has hysteresis.

Figure 7b shows the displacement calculated from the strain measured on 25 November 2020. The maximum displacement occurred at section C, exceeding 6 mm, followed by Section A (4.5 mm), and B (3.0 mm). This means that the most dangerous part is the slope where the MUT crosses the river. The main cause of settlement includes water erosion and MUT sliding. In soils near the water level, water flow includes seepage in the saturated zone and infiltration in the unsaturated zone. Steady Darcy flow in the saturated zone has little effect on soil deformation, but water infiltration in the unsaturated zone has a significant effect on deformation, especially when the first infiltration occurs in the new filled foundation. When soil moisture increases, the hydration film around the particles becomes thicker and the friction between particles decreases, causing fine particles to enter the voids formed by larger particles’ deposition. Therefore, the foundation settlement occurs, with the appearance of the cavity beneath the MUT. In addition, it can also be seen from Figure 7b that the MUTs at positions A and C are easily able to slide toward the bottom of the riverbed under the action of gravity. When the interface between the MUT and soil is dry, the static friction is large enough to prevent sliding. However, when the groundwater level rises and the interface is below the groundwater table, the friction will be greatly reduced, causing the slide of the MUT.

**Figure 8:**
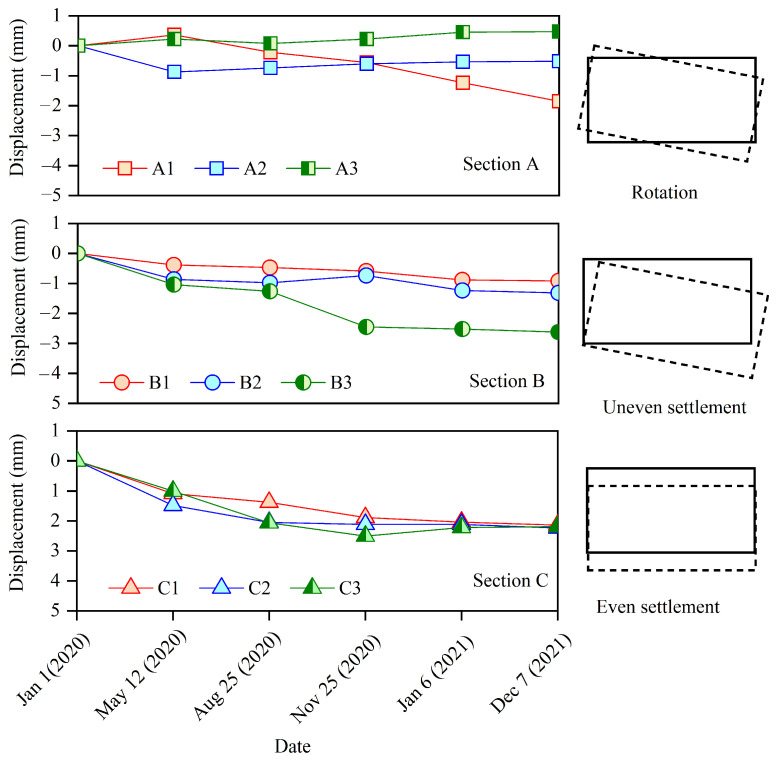
Displacement recorded by the dislocation meters between adjacent MUTs at the positions of sections A, B, and C. The solid line represents the position of MUT before deformation, and the dotted line represents the position after deformation.

**Figure 9:**
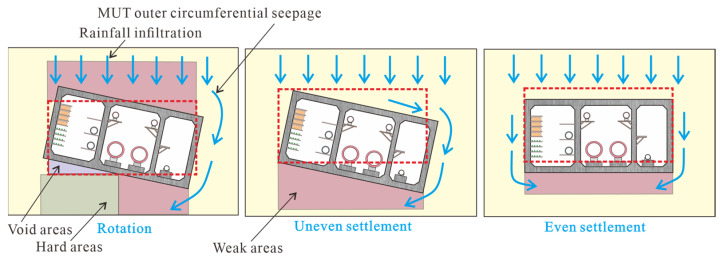
Schematic diagram of the mechanism of rainfall infiltration on MUT settlement. The red dashed box indicates the position of the MUT before deformation.

Figure 8 and Figure 9 show the displacement at the joints recorded by dislocation meters in sections A, B, and C, which is primarily used to analyze the posture of the MUT after settlement. There are three types of MUT damage caused by foundation settlement. When the foundation settles even over a large area, even settlement also occurs when the MUT is laid horizontally, such as that in section C. The typical feature of uniform settlement is that the displacement at each point of the MUT is equal. When the foundation strength is low and settlement occurs on a large scale on one side, the MUT will be accompanied by uneven settlement, as shown in section B in Figure 8. The characteristic of uneven settlement is that the settlement on both sides of the MUT is significantly different. When the lower part of the foundation is uneven, one side is soft and the other side is hard, and the overlying soil pressure is small, so the MUT is prone to rotating, as shown in section A in Figure 8 and Figure 9. There are three reasons for MUT settlement in this case. The first is that loess collapses when it encounters water, resulting in foundation compaction. The second is that soil creeps after the water content exceeds the liquid limit. The third is that soil particles are washed away to form a cavity, which needs to be further detected and determined with the help of GPR technology.

Water infiltration in loess can be divided into unsaturated infiltration and dominant channel infiltration, and the dominant channel increases soil permeability which will lead to the overall downward movement of the wetting [27]. Wang et al. [28] found that the impact of water seepage on the structure and strength of the foundation soil is controlled by the location and the properties of the soil itself. The lower the stress is, the easier it is for seepage channels to form, and the easier it is for the soil to break down [28]. The entire soil deformation and collapse stages include stability, vertical water infiltration, continuous flow, solid particle collapse, mud and water mixture, vault collapse, throughgoing damage, and water–mud gushing [28]. The presence of the MUT alters the original water seepage path, creating a circumferential flow around it [29], as shown in Figure 9. Circumferential flow is controlled by water head pressure, porosity, and soil strength.

### 3.2. MUT Settlement and Deformation Reasons Analysis Based on GPR Detection

**Figure 10:**
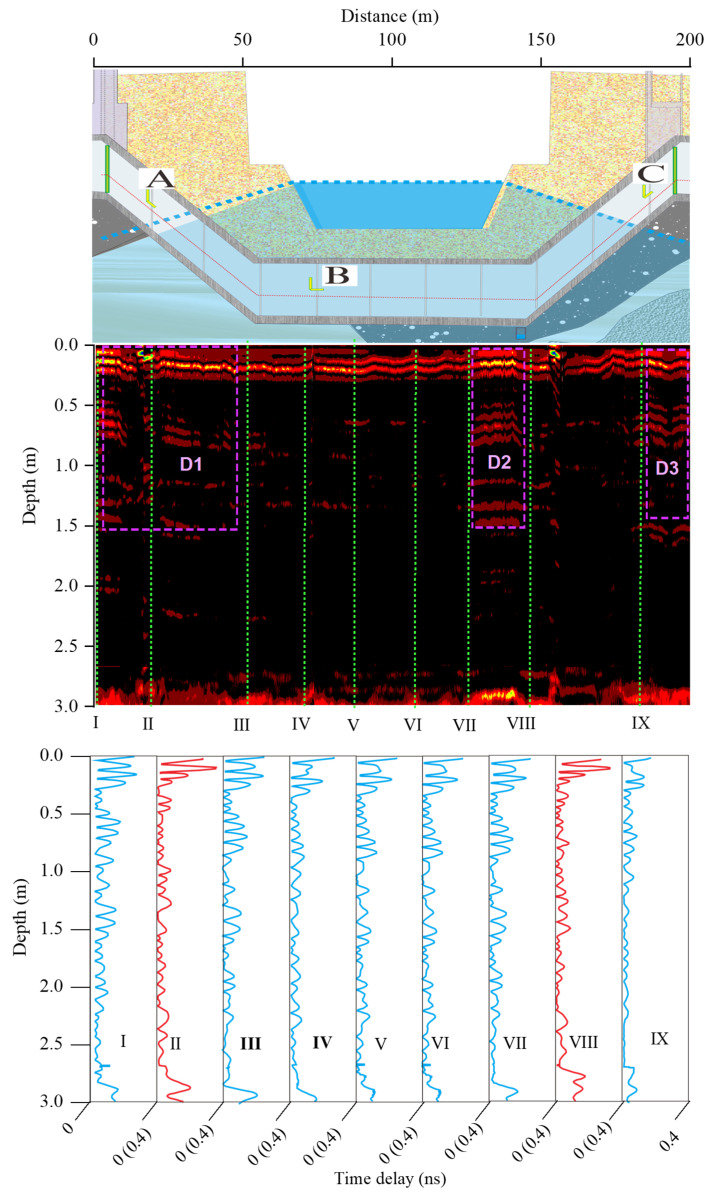
Data of 400 MHz GPR field test. A, B, and C are three cross-sections where dislocation meters are installed. The red time delay curves are located in the D1, D2 and D3 anomaly areas, and the curves in other regions are indicated in blue.

The creep of soil layers under MUT pressure will not produce cavities, while the foundation settlement caused by loess wetting collapse and particle erosion can form cavities or delamination cracks. Therefore, the actual causes for the deformation and settlement must be further determined by GPR images. Figure 10 shows the data obtained by GPR across the King River with a frequency of 400 MHz. The underground voids include lamellar delamination cracks and elliptical cavities. The geological radar image of a delamination crack is parallel to its true shape, which is a straight line segment, while the geological radar reflection time delay curve of the cavity is a hyperbola [24,30,31]. As can be seen from Figure 10, the abnormal area includes three areas, numbered D1, D2, and D3, respectively. No obvious hyperbolic emission signal is observed in Figure 10, so it can be considered that there is no large cavity in the ellipsoid pile. Therefore, the stripe-shaped reflection signal abnormal area can be considered to be caused by delamination.

The lower strata of D1, D2, and D3 include interlayered sandstone, mudstone, and breccia with loess. The geological survey reports indicate that both the mudstone and sandstone are mud–calcium cementation, with horizontal layered structures, and easily softened when exposed to water. In addition, weathering cracks form vertical water channels. Therefore, it can be inferred that the initial delamination cracks were caused by weathering and rapidly enlarged after soaking. It should be emphasized that the construction of the MUT can significantly change the original seepage field in the strata. The original horizontal water flow in the coarse-grained stratum will form a circular flow due to the MUT obstruction, which will accelerate the seepage velocity and scouring at the bottom and top of the MUT [32,33]. Therefore, the cracks between the MUT and the foundation soil are caused by both the accumulated settlement of the lower strata and the scouring effect of the flow around the MUT. D1 and D3 are located directly below sections A and C, respectively. Therefore, the interlayer cracks’ appearance and accumulation caused by water flow are the main controlling factors for the deformation of the upper MUT.

No obvious cracks were observed in the foundation below profile B, so the deformation of the upper MUT was caused by soil creep or collapsible settlement. When the MUT deformation exceeds the warning value and needs to be reinforced or repaired, there are different foundation reinforcement measures for MUT deformation caused by different types of disasters. When obvious voids or interlayer delamination appear in the stratum, filling and reinforcement with low permeability filling materials are required [34,35]. When there are no large voids in the foundation soil and settlement deformation occurs only due to soil structure damage, it is necessary to use highly permeable materials to reinforce the soil structure as a whole [34,35]. Actually, it is difficult to completely avoid deformation and cracks in the lower foundation of MUT; therefore, the main task of managers and maintainers is to control them within a safe range. The influence of delamination cracks’ size and position on MUT deformation needs to be further quantitatively analyzed by combining numerical simulations.

## 4. Conclusions

The MUT in Baiyin City, Gansu Province, China was researched using Brillouin frequency domain analysis (BOFDA), fiber Bragg grating (FBG), and ground penetrating radar (GPR) technologies. The following conclusions can be drawn:

(i) The MUT deformation is mainly caused by joint dislocation. The adjacent MUT joint dislocation caused by foundation settlement can be accurately detected by BOFDA. The dislocation development is determined by intermittent river recharge and water infiltration into the foundation. The maximum strain is prone to appearing at the slopes near the riverbed edges.
(ii) After the deformation position is accurately located by BOFDA, the MUT posture can be judged by the FBG monitored data. Rotation, even settlement, and uneven settlement can be observed through the three FBG-based dislocation meters installed on the top and side walls.
(iii) The cause of MUT settlement is the compression of mudstone and collapse of loess. The interlayer delamination cracks caused by the uncoordinated settlement of the strata and the accumulated cracks at the bottom of the MUT can be accurately located by GPR.

## Figures and Tables

**Table 1:** Strain development of sections A, B, and C measured by SSC.

Sections	Strain (×10^−6^)			
	16 May 2020	25 August 2020	25 November 2020	6 January 2021
Section A	−749	−1048	−2120	−4478
Section B	−722	−85	−320	−3179
Section C	−2178	−3659	−7147	−6838

## Data Availability

The data presented in this study are available on request from the corresponding author.

## Abbreviations

The following abbreviations are used in this manuscript:

BOFDA	Brillouin frequency domain analysis
DFOS	Distributed fiber optic sensing
FBG	Fiber Bragg grating
GPR	Ground penetrating radar
MUT	Multi-purpose utility tunnel
